# Experimental Infection of Rats with Influenza A Viruses: Implications for Murine Rodents in Influenza A Virus Ecology

**DOI:** 10.3390/v17040495

**Published:** 2025-03-29

**Authors:** Long Li, Rirong Chen, Zhigang Yan, Qinglong Cai, Yi Guan, Huachen Zhu

**Affiliations:** 1 Guangdong-Hong Kong Joint Laboratory of Emerging Infectious Diseases, Joint Laboratory for International Collaboration in Virology and Emerging Infectious Diseases (Key Laboratory of Ministry of Education), Joint Institute of Virology (Shantou University-The University of Hong Kong), Shantou University Medical College, Shantou University, Shantou 515063, China; 2 State Key Laboratory of Emerging Infectious Diseases (SKLEID), School of Public Health, Li Ka Shing Faculty of Medicine, The University of Hong Kong, Hong Kong SAR, China

**Keywords:** zoonotic influenza, host range, interspecies transmission, sialic acid receptor, surveillance

## Abstract

*Rattus norvegicus* (brown rat), a widely distributed rodent and common biomedical model, is a known reservoir for many zoonotic pathogens but has not been traditionally recognized as a host for influenza A virus (IAV). To evaluate their susceptibility, we intranasally inoculated Sprague-Dawley rats with various IAV subtypes, including H5Nx, H7N9, H9N2, H10N8 and the 2009 pandemic H1N1. All strains productively infected the rats, inducing seroconversion without overt clinical signs. While replication efficiency varied, all viruses caused significant lung injury with a preferential tropism for the upper respiratory tract. Investigation of receptor distribution revealed a predominance of α2,3-linked sialic acid (SA) in the nasal turbinates and trachea, whereas α2,6-linked SA was more abundant in the lungs. Notably, both receptor types coexisted throughout the respiratory tract, aligning with the observed tissue-specific replication patterns and broad viral infectivity. These findings demonstrate that rats are permissive hosts for multiple IAV subtypes, challenging their exclusion from IAV ecology. The asymptomatic yet pathogenic nature of infection, combined with the global synanthropy of rats, underscores their potential role as cryptic reservoirs in viral maintenance and transmission. This study highlights the need for expanded surveillance of rodents in influenza ecology to mitigate zoonotic risks.

## 1. Introduction

In recent decades, numerous influenza A viruses (IAVs) of zoonotic origin have emerged, causing significant epidemics and even pandemics in humans. Notable examples include the swine-origin 2009 H1N1 pandemic and avian-origin outbreaks such as H7N9, H10N8, H9N2, and highly pathogenic avian influenza (HPAI) H5Nx [1,2,3]. These events have repeatedly underscored the critical ecological role of animals in the genesis and transmission of IAVs.

IAVs exhibit a broad host range, with frequent interspecies spillover events observed in nature settings. Historically, IAVs have established endemic circulation in birds, bats, horses, pigs, dogs, and humans [4,5]. Sporadic infections have also been reported in diverse ungulates and carnivores, including camels, dairy cows, whales, cats, and mink [4,5,6,7,8,9]. While wild waterfowl are recognized as the primary natural reservoir for IAVs, domestic animals—both avian and mammalian species—serve as critical intermediate hosts, facilitating viral adaptation and the emergence of novel human-infective variants [4,5]. Consequently, monitoring IAVs in diverse animal species and advancing our understanding of influenza virus ecology are imperative for mitigating zoonotic risks.

Rodents, including commensal rats, are known carriers of many zoonotic pathogens but are not generally regarded as natural hosts of IAVs [4,5,10,11]. However, the detection of IAV nucleic acids and antibodies against viruses such as HPAI H5N1 and H5N6 in rodents suggests potential field exposure [12,13,14,15,16,17]. While laboratory mice (*Mus musculus*) and rats (*Rattus norvegicus*) are widely used in biomedical research, and mice are commonly employed in influenza studies, rats have not been considered susceptible hosts or ideal models for most IAVs. Reports on influenza infection in experimental *Rattus* species are limited, often involving adapted viral strains [18,19,20], infant rat models [18,21,22,23], or other non-generalizable conditions [24,25]. Historically, researchers from Russia and other countries have used white rats for the production of polyclonal antisera, influenza vaccine development, and immunotoxicity studies [26,27,28], but these typically involved intraperitoneal injection or other immunization methods rather than mucosal inoculation.

In this study, we experimentally infected rats with different subtypes of IAVs isolated from the field, focusing on prevalent lineages with zoonotic potential. Our aim is to provide direct evidence of the susceptibility of rats to IAVs without prior viral adaptation. As common synanthropes worldwide, rats are ubiquitous in both rural and urban areas with dense populations [29,30]. They share habitats with wild and free-ranging animals and have unrestricted access to human settlements and livestock facilities. Understanding the role of rats in IAV ecology is critical for risk assessment and the development of strategies for disease control and prevention.

## 2. Materials and Methods

### 2.1. Ethical Statement

All animal experiments were conducted in compliance with institutional guidelines and ethical standards. The study design and procedures were approved by the Medical Animal Care and Welfare Committee of Shantou University Medical College (Reference Nos. SUMC2013-111 and SUMC2016-034). Experiments involving infectious agents were performed in Biosafety Level 3 (BSL-3) facilities. The research adhered to the Guangdong Provincial Regulations on the Administration of Laboratory Animals and followed the Operational Guidelines for Ethics Committees that Review Biomedical Research issued by the Ministry of Health of China.

### 2.2. Viruses

Viruses of prevalent and recently emerging subtypes in China were selected for this study. Viral stocks were prepared based on their original hosts, regardless of prior culturing history: avian strains were passaged in 9-day-old embryonated chicken eggs, while human isolates were propagated in Madin-Darby canine kidney (MDCK) cells [31]. Aliquots of the viral stocks were titrated using the 50% tissue culture infectious dose (TCID_50_) method and stored at −80 °C until use. The representative strains used in this study are summarized in Table 1.

### 2.3. Animal Experiments

Female specific-pathogen-free (SPF) Sprague-Dawley (SD) rats, aged 8 to 9 weeks, were purchased from Beijing Vital River Laboratory Animals Co., Ltd. (Beijing, China) and randomly divided into groups. Animals were anesthetized by intramuscular injection of sodium pentobarbital (0.2 mg/g) prior to inoculation and necropsy. Rats that lost more than 30% of their initial body weight were humanely euthanized, and respiratory tissues and blood were collected where possible.

To assess the infectivity of different IAV subtypes in SD rats, two groups of three rats and one group of eight rats were inoculated with 100 μL of phosphate-buffered saline (PBS) containing 10^2^, 10^4^ and 10^6^ TCID_50_ of each virus strain, respectively. Eight rats were sham-infected with 100 μL PBS as controls. Five rats in the 10^6^ TCID_50_ dose group were monitored for 14 days and compared to the control group (n = 5), while the remaining rats were necropsied at 4 days post-inoculation (dpi) for respiratory tissues collection. At 4 dpi, three rats in each virus dose group were euthanized. Each tissue was divided: one portion was homogenized in PBS, stored at −80 °C, and titrated on MDCK cells, while the remainder was fixed in 10% neutral buffered formalin for histopathological examination. Blood was also collected for serum preparation.

### 2.4. Hemagglutination Inhibition Test

Seroconversion was assessed at 4 or 14 dpi post-euthanasia. Rat serum was treated with receptor-destroying enzyme II (RDE II; Denka Seiken, Tokyo, Japan) overnight at 37 °C, heat-inactivated at 56 °C for 30 min, and adsorbed with turkey red blood cells (TRBCs) to remove non-specific agglutinins. Hemagglutination inhibition (HAI) assays were performed using the IAVs listed in Table 1, following the standard protocol of the World Health Organization (WHO) [31]. Briefly, four hemagglutination units of each virus were mixed with serially diluted sera in U-bottom 96-well plates and incubated for 45–60 min. TRBCs (0.5%) were added, and plates were incubated for 30 min at room temperature. HAI titers were defined as the reciprocal of the highest serum dilution that inhibited hemagglutination.

### 2.5. Immunohistochemical and Histopathological Studies

Nasal, tracheal and lung tissues were fixed in 10% (vol/vol) neutral buffered formalin for 48 h, dehydrated in graded ethanol and xylene, and embedded in paraffin. Sections (3 μm) were cut using a Leica slicer and subjected to immunohistochemical (IHC) staining for viral nucleoprotein (NP) detection and hematoxylin and eosin (H&E) staining for histopathological examination, as previously described [32,33].

### 2.6. Lectin Staining Assay

Sialic acid (SA) receptor expression in rat airways was determined using biotinylated lectins: *Maackia amurensis* agglutinin I and II (MAA I and MAA II; Vector Laboratories, Burlingame, California, USA) for α2,3-linked SA, and *Sambucus nigra* agglutinin (SNA; Vector Laboratories) for α2,6-linked SA. MAA I and II specifically bind SAα2,3Galβ1,4GalNAc and SAα2,3Galβ1,3GalNAc, respectively, while SNA preferentially binds α2,6-linked SA, with weaker affinity for α2,3-linked SA [34,35,36]. Tissues from non-inoculated SPF SD rats were processed as described for IHC and histopathological studies. Sections were incubated with MAA (1:1500) or SNA (1:2000) for 1 h at room temperature. For comparison, the SA receptor distribution was also analyzed in 8- to 9-week-old female C57BL/6 mice (Beijing Vital River Laboratory Animals Co., Ltd., Beijing, China).

### 2.7. Statistical Analysis

Statistical analyses were performed using GraphPad Prism 10.1.0 (GraphPad Software). Data are presented as mean ± standard deviation unless otherwise stated. Student’s *t*-test was used for two-group comparisons, and one-way analysis of variance (ANOVA) was used for multiple-group comparisons. Statistical significance was set at *p* < 0.05.

## 3. Results

### 3.1. Asymptomatic Infection with IAVs in SD Rats

**Figure 1:**
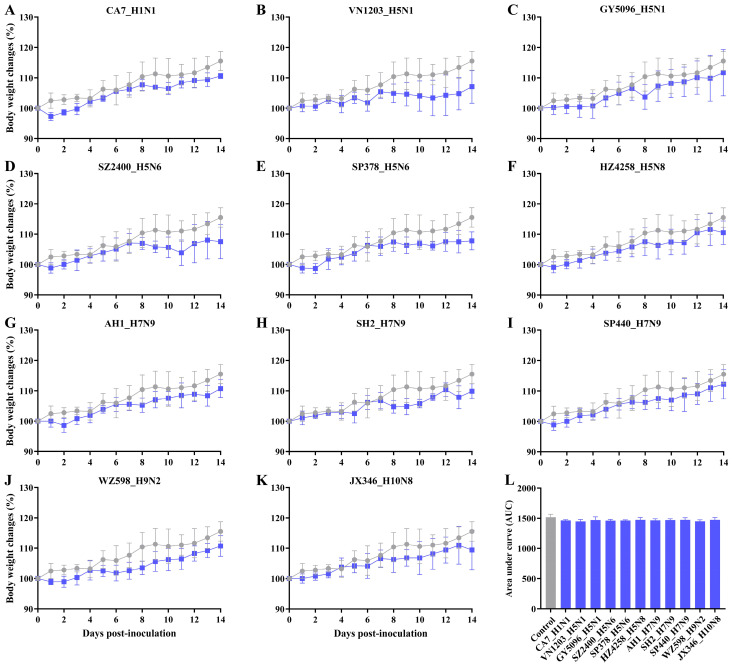
Changes in body weight of Sprague-Dawley (SD) rats inoculated with selected influenza A viruses (see Table 1). To evaluate the pathogenicity of different influenza viruses in rats, 8- to 9-week-old SD rats were inoculated with 10^6^ TCID_50_ of the indicated viruses (**A**–**K**). Body weight was monitored daily for 14 days post-inoculation (dpi). No deaths occurred in any group during the observation period. Data present mean changes in body weight ± standard deviation for five rats in the control group (grey) and five rats in each virus-inoculated group (blue). The area under the curve (AUC) for each group was calculated using GraphPad Prism 10.1.0 (**L**). One-way analysis of variance (ANOVA) of the group-specific AUC and pairwise t-tests between the virus-inoculated groups and the control group revealed no statistically significant changes in body weight (*p* > 0.05).

No clinical signs, such as ruffled hair, inactivity, reduced food and water intake, or other influenza-like illness, were observed in any of the rats. Body weight in the control group increased steadily, and no deaths occurred in any inoculated groups (Figure 1). Following inoculation with different IAV subtypes, rats in each group either maintained their weight or experienced a slight weight loss of less than 5% within the first one or two days, followed by weight gain over the observation period. Notably, some HPAI H5 strains (GY5096 H5N1, SZ2400 H5N6 and HZ4258 H5N8) induced a brief weight loss at 8 or 9 dpi, occurring after a 5- or 6-day period of weight gain (Figure 1C,D,F). In contrast, rats in the other groups exhibited a stable rate of weight gain over the 14-day period. However, compared to mock-inoculated controls, none of the virus-inoculated groups showed significant changes in body weight during the infection (Figure 1L, *p* > 0.05). These findings indicate that infection with either endemic or emerging IAVs, including HPAI H5 and HPAI H7 viruses, does not cause overt morbidity in the SD rat model, despite subtle and non-significant impairment in weight gain.

### 3.2. Replication and Distribution of Different IAVs in the Rat Respiratory Tract

**Figure 2:**
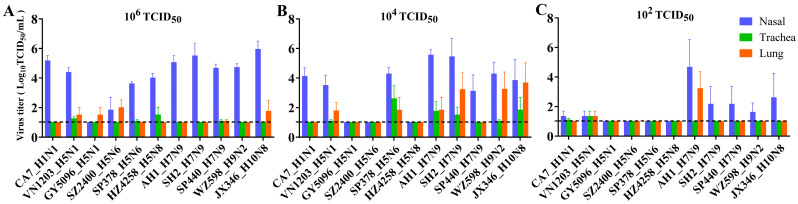
Replication of selected influenza A viruses in the respiratory tract of SD rats. To evaluate the infectivity of different influenza viruses (see Table 1) in rats, 8- to 9-week-old SD rats were inoculated with doses of 10^6^ (**A**), 10^4^ (**B**), and 10^2^ (**C**) TCID_50_ of the indicated viruses. Animals were humanely euthanized, and tissues harvested for virus titration 4 days post-inoculation. Virus titers in the nasal turbinate (blue), trachea (green), and lung (red) were determined by titration on MDCK cells, and TCID_50_ was calculated using the Reed-Muench method. Log_10_TCID_50_/mL is shown, with error bars representing standard deviations for three individual rats in each virus group. No viable virus was detected in the control group.

To assess viral replication in the rat respiratory tract, groups of three rats were inoculated with varying doses (10^2^, 10^4^, and 10^6^ TCID_50_) of each virus and necropsied at 4 dpi to determine viral presence and titers. At the 10^2^ TCID_50_ dose, limited replication of CA7, the pandemic 2009 H1N1 (pdmH1N1) prototype virus, was detected in the nasal turbinate and trachea (Figure 2C). Nasal viral replication increased to 10^4^ TCID_50_/mL and 10^5^ TCID_50_/mL in groups challenged with 10^4^ TCID_50_ and 10^6^ TCID_50_ doses of CA7, respectively (Figure 2A,B). Among the HPAI H5 viruses, only VN1203 (H5N1 clade 1.0) was detected in the respiratory tract after inoculation with the low 10^2^ TCID_50_ dose, with replication titers below 10^2^ TCID_50_/mL (Figure 2C). At the 10^4^ TCID_50_ dose, only VN1203 (H5N1) and SP378 (H5N6 with internal genes derived from H9N2) were detected in the airways (Figure 2B). These two viruses replicated at moderate levels, with the highest titers observed in the nasal turbinates. All HPAI H5 viruses were detected after inoculation with the 10^6^ TCID_50_ dose (Figure 2A), but nasal titers in the H5 groups were generally lower compared to other subtypes. For H7N9, AH1 (LPAI prototype H7N9) replicated to over 10^4^ TCID_50_/mL in the nasal turbinate of the 10^2^ TCID_50_ group, approximately two logs higher than SH2 (LPAI H7N9) and SP440 (HPAI H7N9), and was also detected in the lungs (Figure 2C). At higher doses, nasal titers for SH2 and SP440 increased, approaching the level of AH1 (Figure 2A,B). WZ598 (H9N2) and JX346 (H10N8) displayed replicative capacity comparable to SH2 (H7N9) or SP440 (H7N9) at the same doses, with nasal titers also increasing with higher inoculation doses (Figure 2A–C). In summary, for most viruses tested, titers were significantly higher in the nasal turbinate than in the trachea and lung, indicating a preference for replication in the upper respiratory tract of rats.

**Figure 3:**
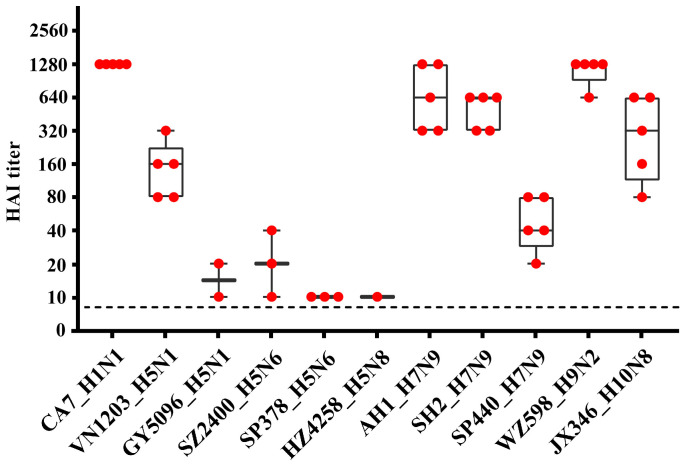
Serological antibody titers in rats challenged with selected influenza A viruses. Blood samples from rats inoculated with 10^6^ TCID_50_ of each virus (see Table 1) were collected for serological analysis at the end of the 14-day experiment (14 dpi). Antibodies to influenza A viruses were measured using the hemagglutination inhibition (HAI) test. Data from seropositive rats are presented here, with each red dot representing one rat; seronegative (HAI titer < 10) and control group data are provided in Table S1.

Blood samples were collected for serological testing during euthanization. We performed the HAI assay and observed successful seroconversion at 14 dpi after virus challenge in this rat model (Figure 3, Table S1). However, no antibodies were detected in the sera of rats euthanized at 4 dpi. Although rats produced antibodies against HPAI H5 and HPAI H7N9 viruses by 14 dpi, the titers were significantly lower compared to groups infected with other tested viruses. In the HPAI groups, all five rats inoculated with VN1203 (H5N1 clade 1.0) and SP440 (H7N9) became seropositive, with antibody titers ranging from 40 to 320. These titers were lower than those observed for low-pathogenic pdmH1N1, H7N9, H9N2, and H10N8 viruses, which all exceeded 320 in each individual. For the other HPAI H5 virus groups (i.e., clade 2.3.4.4 virus groups), some animals remained seronegative, and antibody titers in seropositive rats were low or marginal, ranging from 10 to 40.

Overall, our findings demonstrated that the tested viruses were capable of infecting rats without prior adaptation and replicated productively in this species. However, antibody production levels varied significantly among strains.

### 3.3. Pneumonia and Histopathological Changes Induced by IAVs in SD Rats

**Figure 4:**
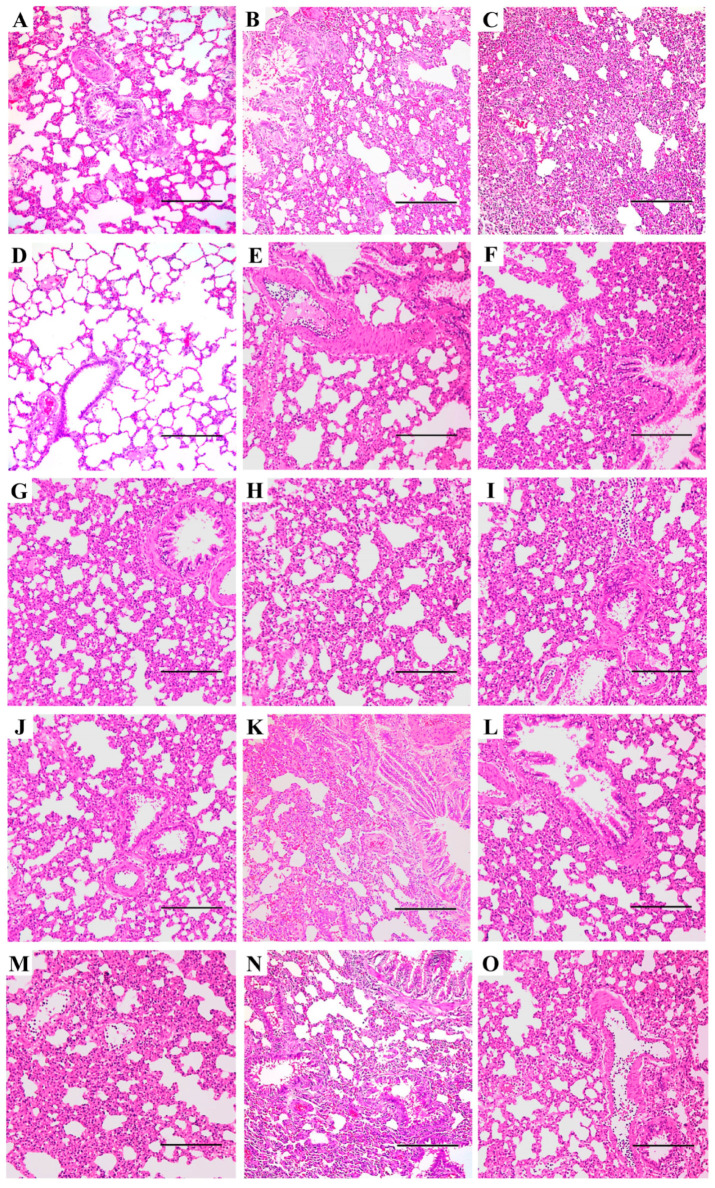
Histopathological analysis of the lungs of SD rats inoculated with different subtypes of influenza A virus. Three rats per virus group were inoculated at the indicated doses, and lungs were harvested at 4 dpi. Hematoxylin-eosin (H&E) staining was performed on 3 μm sections. Whole sections were used for analysis, but only representative images are shown, as pathological changes were comparable among the different virus groups at the same dose. Dose-dependent effects are illustrated with representative lung sections from rats inoculated with SH2 (H7N9) at 10^2^ TCID_50_ (**A**), 10^4^ TCID_50_ (**B**), and 10^6^ TCID_50_ (**C**). Pathological changes in the lungs of mock-infected SD rats (**D**) and those inoculated with all tested influenza A viruses are shown with representative lung sections from the 10^4^ TCID_50_ group ((**E**), CA7, pandemic 2009 H1N1; (**F**), VN1203, H5N1; (**G**), GY5096, H5N1; (**H**), SZ2400, H5N6; (**I**), SP378, H5N6; (**J**), HZ4258, H5N8; (**K**), AH1, H7N9; (**L**), SH2, H7N9; (**M**), SP440, H7N9; (**N**), WZ598, H9N2; (**O**), JX346, H10N8). Scale bars indicate 200 μm.

Pneumonia was not observed in the control group (Figure 4D). Although the gross view of excised lungs appeared normal, with no visible lesions in inoculated rats, H&E staining revealed significant lung injury and comparable pathological changes among the different virus groups at the same dose (Figure 4B,E–O). However, the severity of these changes varied between the different doses (Figure 4A–C). Virus-infected rat lungs exhibited typical histopathological changes compared to mock controls. Alveolitis, widened alveolar septum, inflammatory infiltration, and erythrocyte extravasation were commonly observed across all three doses (Figure 4). At the 10^2^ TCID_50_ dose, pneumonia was relatively mild, with intact alveolar cells still observable, but obvious widening of the alveolar septum was present (Figure 4A). In the higher dose groups (10^4^ and 10^6^ TCID_50_), alveolar cells were destroyed, and marked exudation due to inflammation was observed (Figure 4B,C,E–O). Alveolar fusion, markedly thickened alveolar walls, necrosis, and loss of bronchial epithelial cells were also evident (Figure 4). Rats inoculated with 10^6^ TCID_50_ IAVs displayed the most severe pneumonia, with pulmonary consolidation occurring in most parts of the lung. This was characterized by complete filling of the alveolar space with exudates and inflammatory factors, replacing alveolar air (Figure 4C). To provide a comprehensive overview of IAV-induced pneumonia in rats, representative pathological changes in the lungs of the 10^4^ TCID_50_ groups for all viruses are shown (Figure 4E–O). Collectively, all tested IAVs elicited an inflammatory response and lung injury in SD rats, indicating productive infection and pathogenicity caused by these viruses.

**Figure 5:**
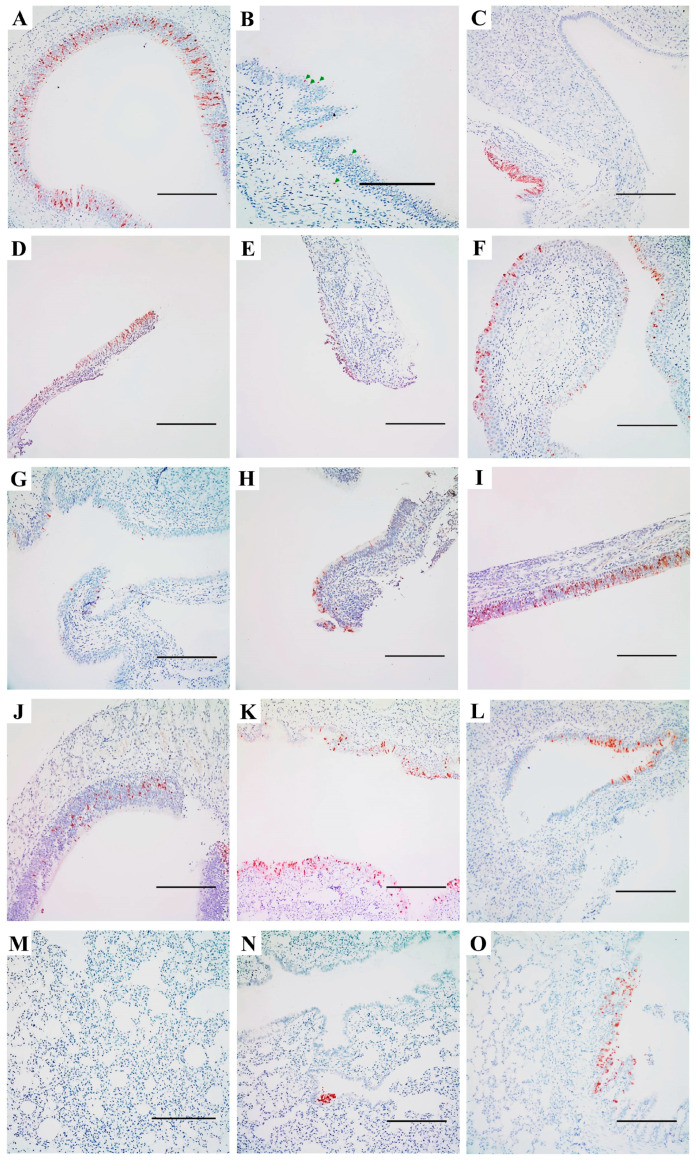
Immunohistochemical analysis of influenza virus nucleoprotein (NP) in the respiratory tract of SD rats. Airway tissues were harvested at 4 dpi and 3 μm sections were used for immunohistochemical analysis. A representative image was selected for viruses with similar virus distribution patterns. Influenza NP antigens (brown) are shown in the nasal turbinate ((**A**), CA7, pandemic 2009 H1N1, 10^6^ TCID_50_; (**B**), HZ4258, H5N8, 10^6^ TCID_50_, with arrows indicating the positively stained cells; (**C**), AH1, H7N9, 10^2^ TCID_50_; (**D**), AH1, H7N9, 10^4^ TCID_50_; (**E**), SH2, H7N9, 10^2^ TCID_50_; (**F**), SH2, H7N9, 10^4^ TCID_50_; (**G**), SH2, H7N9, 10^6^ TCID_50_; (**H**), SP440, H7N9, 10^2^ TCID_50_; (**I**), SP440, H7N9, 10^4^ TCID_50_; (**J**), SP440, H7N9, 10^6^ TCID_50_; (**K**), WZ598, H9N2, 10^6^ TCID_50_; (**L**), JX346, H10N8, 10^4^ TCID_50_) and lungs ((**M**), VN1203, H5N1, 10^4^ TCID_50_; (**N**), AH1, H7N9, 10^2^ TCID_50_; (**O**), WZ598, H9N2, 10^4^ TCID_50_). Scale bars indicate 200 μm.

To confirm productive infection and the presence of IAVs in the respiratory tract of SD rats, IHC staining was performed to detect viral NP antigen in nasal turbinate and lung tissues. Significant differences in NP distribution were observed between viruses. In the nasal turbinate, substantial NP antigen from pdmH1N1 was detected in rats inoculated with CA7 at the 10^6^ TCID_50_ dose (Figure 5A). Among the five HPAI H5 virus strains, only a few NP proteins from the HZ4258 (H5N8) group were detected in one rat from the 10^6^ group (Figure 5B, green arrows). In contrast, NP antigens of all H7N9 strains were detected in the nasal turbinate after inoculation (Figure 5C–J). AH1 (LPAI H7N9) antigens were detected in the nasal turbinate of the 10^2^ and 10^4^ TCID_50_ groups (Figure 5C,D) but not in the 10^6^ TCID_50_ group. For the other two H7N9 viruses, SH2 (LPAI H7N9) and SP440 (HPAI H7N9), viral antigens were present in all three dose groups, as shown in Figure 5E–G and Figure 5H–J, respectively. In the remaining groups, viral NP from WZ598 (H9N2) and JX346 (H10N8) was detected in nasal tissues from the 10^6^ TCID_50_ group (Figure 5K) and the 10^4^ TCID_50_ group (Figure 5L), respectively. In contrast, viral NP staining was negative in the lungs of most groups (Figure 5M). Only NP antigens from WZ598 (H9N2) and AH1 (H7N9) were detected in single rats from each group (Figure 5N,O). In the H9N2-positive lung section, bronchiolar epithelial cells were focally stained positive for WZ598 in one rat from the 10^4^ TCID_50_ group (Figure 5O). For H7N9, stained antigens were observed only in the lung of one rat inoculated with 10^2^ TCID_50_ of AH1 (Figure 5N), despite previous detection of H7N9 strains in lung tissues by virus titration (Figure 2B). In NP-positive nasal and lung sections, viral NP was primarily localized in ciliated mucosal epithelial cells.

Collectively, the IHC staining results revealed a predominant distribution of viral NPs in the rat nasal cavity, consistent with the replication pattern of higher viral titers in the nasal turbinate compared to the lungs. These findings suggest that the tested IAVs preferentially replicate in the upper respiratory tracts of SD rats, providing further evidence of viral replication and pathogenesis.

### 3.4. Expression of Sialic Acid-Linked Receptors in the Respiratory Tract of SD Rats

**Figure 6:**
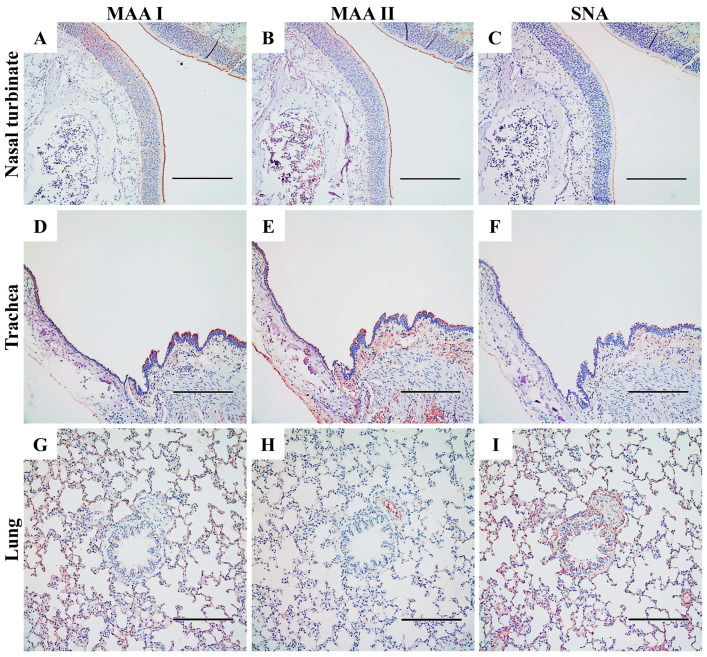
Distribution of α2,3- and α2,6-linked sialosides in the respiratory tracts of SD rats. The presence of α2,3- and α2,6-linked sialosides in the nasal turbinate (**A**–**C**), trachea (**D**–**F**) and lung (**G**–**I**) of naïve rats were detected using biotinylated *Maackia amurensis* agglutinin I or II (MAA I, MAA II) for α2,3-linked sialosides or *Sambucus nigra* agglutinin (SNA) for α2,6-linked sialosides. Both α2,3- and α2,6-linked sialosides are shown in brown. Scale bars indicate 200 μm.

The attachment and entry of IAVs into host cells depend on the presence of specific SA-linked receptors. To investigate the expression of these receptors in the airways of rodents, we first examined the presence of α2,3- and α2,6-linked SA receptors in mice, using positive staining for MAAs and SNA (Figure S1). In contrast to the weak MAA II staining observed in mice (Figure S1B,E,H), SD rats exhibited significantly stronger expression of α2,3-linked SA throughout the upper and lower respiratory tracts (Figure 6B,E,H). This indicates a notable difference in the presence of SAα2,3Galβ1,3GalNAc, the “traditional” avian influenza receptor, between these two rodent species [34].

In the nasal turbinate of SD rats (Figure 6A–C), both MAAs and SNA bound to ciliated epithelial cells, indicating the presence of α2,3- and α2,6-linked SA receptors, respectively. However, MAAs (Figure 6A,B) exhibited much denser staining in these nasal epithelial cells compared to SNA (Figure 6C). Additionally, cells in the basal and connective tissues were exclusively stained by MAAs but not by SNA, suggesting the presence of α2,3-linked SA receptors and the absence of α2,6-linked SA receptors in these areas. In the lower respiratory tract, staining for both MAA isoforms was observed in the trachea of rats (Figure 6D,E) and mice (Figure S1D,E). However, SNA-positive staining was less abundant in the epithelial cells of the rat trachea (Figure 6F) compared to MAAs, and minimal staining was noted in mice (Figure S1F). In the lungs of SD rats (Figure 6G–I), α2,6-linked SA receptors were more abundant in the bronchi and bronchioles, as indicated by denser SNA staining compared to MAAs. In the alveoli, the staining patterns of MAA I and SNA were similar due to their diffuse distribution, although MAA II staining was significantly reduced. These results demonstrate that α2,3-linked SA receptors are predominantly expressed in the nasal turbinate, trachea, and alveoli, whereas α2,6-linked SA receptors are more abundant in the lungs of SD rats. This differential expression may provide insight into the mechanisms underlying the variable replication and transmission of IAVs in this model.

## 4. Discussion

The role of rats and other rodents in influenza ecology remains understudied and controversial. This study provides compelling experimental evidence that SD rats, a representative model of the *Rattus* species, are susceptible to productive infection by diverse subtypes of contemporary IAVs that pose significant threats to both public health and agriculture. These include avian HPAI H5Nx (clades 1.0 and 2.3.4.4a, b, e, g, both human and avian isolates), H7N9 (HPAI and LPAI), H9N2, H10N8, and the mammalian-adapted pandemic 2009 H1N1 viruses (Table 1). Notably, these infections occurred without prior viral adaptation, challenging the conventional assumptions that rats are generally insusceptible and not natural hosts of IAV. Our findings, together with previous reports [11,12,13,14,15,16,17,37,38,39,40], underscore the need to reevaluate rodents as potential reservoirs, mechanical vectors, or bridging hosts in the zoonotic transmission of IAVs. The absence of overt clinical signs, despite robust viral replication, seroconversion, and histopathological evidence of lung injury (Figure 1, Figure 2, Figure 3, Figure 4 and Figure 5), positions rats as cryptic carriers capable of sustaining IAV infections undetected in natural settings.

A striking feature of IAV-infected SD rats is the dissociation between their subclinical manifestations and significant virological and immunological findings. Unlike mice and ferrets, which develop observable disease or mortality following experimental IAV challenge [32,33], rats exhibited only a statistically insignificant lower rate of weight gain compared to the control group (Figure 1) and no influenza-like symptoms, even when infected with HPAI H5 or H7N9 strains. This asymptomatic phenotype resembles that of wild waterfowl, the natural reservoirs of IAV [4,5]. The absence of disease presentation and the induction of seroconversion in most rats suggests that rats may use effective immune mechanisms to limit systemic viral spread while allowing local replication in the upper respiratory tract (this study and [19,20,37]). This balance may facilitate viral infection without compromising host survival, positioning rats as potential stealth vectors in ecosystems where they interact with domestic animals, wildlife, and humans.

The receptor distribution landscape has been previously reported in BABL/c and C57BL/6J mice and in mouse tracheal epithelial cells (mTEDCs) [34,36]. To our knowledge, here we provide the first report on the distribution of α2,3- and α2,6-linked SA in the respiratory tracts of SD rats (Figure 6). The preferential replication of IAVs in the nasal turbinate, as evidenced by higher viral titers and NP antigen localization (Figure 2 and Figure 5), highlights a distinct tissue tropism in rats. This pattern correlates with the predominance of α2,3-linked SA receptors in the upper respiratory tract (Figure 6), which are preferentially recognized by avian-adapted IAVs. However, the concurrent detection of both α2,3- and α2,6-linked SA receptors in the rat lung suggests dual receptor expression that could permit infection of the lower respiratory tract, as observed with H7N9, H9N2, H10N8 and some H5 strains, such as the human isolates VN1203 and SP378 (Figure 2). The discordance between the SA receptor distribution (α2,6-SA dominance in the lung with MAA I-positive staining of α2,3-SA) and the relatively low pulmonary viral titers (especially for the mammalian-adapted strains like pdmH1N1) implies that additional host factors, such as mucin barriers, antiviral interferon responses, protease availability, and other unknown mechanisms may restrict systemic dissemination. This receptor-replication mismatch warrants further investigation and caution when extrapolating murine data to other rodents, particularly given the contrasting SA expression profiles in mice, which exhibit much weaker α2,3-SA expression compared to rats.

Although viable virus was detected for all virus strains, our results revealed significant differences in replication efficiency in rats between IAV strains. This disparity may reflect subtype-, clade-, or strain-specific features. Notably, all strains carrying the G57 H9N2-like internal genes (i.e., SP378, AH1, SH2, SP440, WZ598, and JX346) [41,42,43,44,45,46] replicated robustly in the upper respiratory tract of rats. This may indicate a potential growth advantage conferred by this specific internal gene constellation. However, this hypothesis requires further investigation. Of note, the efficient replication of H9N2, an enzootic subtype in Eurasia with a broad host range and pandemic potential, and its derivatives suggests that rats may contribute to the evolution of the related viruses. The predominance of the G57 H9N2 genotype in China [41], which is associated with human infections and the emergence of novel avian strains (such as H7N9/H7N7 [42,43], H10N8/H10N6 [44,45], H5N6 [46] and H3N8 [47]), further underlies this risk, particularly as rats may face increased exposure and susceptibility to infection.

Despite the absence of clinical disease, all IAVs induced significant pulmonary pathology characterized by alveolitis, inflammatory infiltration, and epithelial necrosis (Figure 4). The dose-dependent severity of the lung injury implies the potential for subclinical infections to cause covert tissue damage that could predispose rats to secondary infections or alter their respiratory dynamics in natural settings. Of particular concern is the capacity of clade 2.3.4.4 HPAI H5 viruses to replicate in rats without prior adaptation, albeit with variable viral titers. Although these strains exhibited relatively low nasal titers compared to H7N9 or H9N2 viruses, their detection in the respiratory tract raises questions about the risk of rat-mediated viral persistence or reassortment in regions where HPAI circulates enzootically. Although we have not tested the currently devastating H5N1 clade 2.3.4.4b strains circulating in the Americas, the Animal and Plant Health Inspection Service recently detected this HPAI virus in several black rats (*Rattus rattus*) in Riverside, California, during February 2025, alongside numerous prior reports of infections in deer mice (*Peromyscus maniculatus*) and house mice (*Mus musculus*) across the United States [48]. These findings collectively underscore the ever-expanding host spectrum of HPAI H5N1 and emphasize the underappreciated role of murine rodents in influenza ecology, highlighting the critical need to investigate their capacity to sustain viral transmission in natural settings.

The ubiquity of rats in human-dominated ecosystems amplifies their potential role as intermediaries in IAV transmission cycles. As synanthropes, rats have free access to poultry and livestock farms, wet markets, wildlife, and urban garbage dumps, creating opportunities for cross-species virus exchange. Their preferential replication in the upper respiratory tract and shedding of virus in nasal secretions could facilitate environmental contamination, enabling mechanical transmission to susceptible hosts. Animals that prey on rats or scavenge carcasses, including but not limited to cats, dogs, and other carnivores, may also be infected. Although IAV transmission by rats has not been confirmed, animal infections with IAVs through exposure to contaminated environments via virus-laden fomites and consumption of contaminated food and water have been recorded in cats, chickens, mice, and SD rats, naturally or experimentally [7,38,48,49,50,51,52].

It is also worth noting that the coexistence of α2,3- and α2,6-SA receptors raises the possibility that rats may act as “mixing vessels” for reassortment, particularly when co-infected with avian and human IAVs. While no natural reassortants have been reported in rats, the detection of IAV nucleic acids in wild *Rattus norvegicus* populations (11.04% in Boston, Massachusetts and 3.47% in southern China) [14,15] suggests ongoing spillover events that merit closer surveillance. Furthermore, the asymptomatic nature of infection complicates surveillance efforts, necessitating active molecular or serological screening in wild rat populations to assess their contribution to IAV maintenance.

This study focused on a laboratory rat model under controlled conditions that may not fully replicate the physiological or immunological state of wild rats. Virus replication outside the respiratory tract was not explored. Natural routes of exposure (e.g., oral, aerosol, fomite, etc.) and co-infections with other pathogens could alter infection outcomes. Additionally, the lack of transmission experiments leaves it unresolved whether rats can horizontally transmit IAVs from rat to rat or to the other mammals—a critical question for evaluating their role in viral ecology. While receptor binding specificity has been inferred from previous studies, direct characterization of the viral hemagglutinin affinity for rat SA receptors is also needed to clarify tropism mechanisms. Future studies should prioritize in vivo transmission assays via different routes, comparative receptor profiling in various wild rodents, and investigations into the molecular determinants of viral fitness in rat respiratory tissues. Additional strains could be included in the experiments to further expand our understanding of IAV infections in this animal. Systematic screening of wild rat populations for IAV nucleic acids and antibodies, particularly in regions with enzootic H5, H7, H9, or H10 viruses, is also warranted to assess the role of rats in influenza A virus transmission and evolution.

## 5. Conclusions

This study redefines *Rattus norvegicus* as a permissive host for multiple IAV subtypes prevalent in birds or humans and highlights its ability to sustain subclinical infections with potential ecological consequences. The convergence of broad viral susceptibility, synanthropic behavior, and dual SA receptor expression in the respiratory tracts positions rats as underrecognized players in influenza ecology. While their role as “mixing vessels” remains speculative, the risk of environmental virus amplification and spillover to domestic animals or humans cannot be dismissed. Strengthening surveillance in rodent populations and integrating rats into One Health frameworks will be essential for mitigating zoonotic threats in an era of escalating avian influenza activity.

## Figures and Tables

**Table 1:** Summary of the influenza A virus strains used in this study.

Strain Name	Abbreviation	Subtype	Clade ^1^
A/California/07/2009	CA7	H1N1	H1N1/2009 prototype
A/Vietnam/1203/2004	VN1203	H5N1	HAPI H5 clade 1.0
A/Duck/Guiyang/5096/2013	GY5096	H5N1	HPAI H5 clade 2.3.4.4g
A/Chicken/Shenzhen/2400/2013	SZ2400	H5N6	HPAI H5 clade 2.3.4.4a
A/Shenzhen/SP378/2015	SP378	H5N6	HPAI H5 clade 2.3.4.4e
A/Duck/Huzhou/4258/2013	HZ4258	H5N8	HPAI H5 clade 2.3.4.4b
A/Anhui/1/2013	AH1	H7N9	H7N9 prototype
A/Shanghai/2/2013	SH2	H7N9	H7N9 prototype-like
A/Guangdong/SP440/2017	SP440	H7N9	HPAI H7N9
A/Chicken/Wenzhou/598/2013	WZ598	H9N2	H9N2 Y280 lineage
A/Jiangxi-Donghu/346/2013	JX346	H10N8	H10N8 prototype

^1^ All H5 viruses and the SP440 (H7N9) are highly pathogenic avian influenza (HPAI) strains, while the others are low pathogenic (LPAI).

## Data Availability

The original contributions presented in this study are included in the article and Supplementary Material. Further inquiries can be directed to the corresponding authors.

## Supplementary Materials

The following supporting information can be downloaded at: https://www.mdpi.com/article/10.3390/v17040495/s1, Figure S1: Distribution of α2,3 and α2,6-linked sialosides in the respiratory tracts of mice; Table S1: Seroconversion in Sprague-Dawley (SD) rats inoculated with the tested influenza A viruses.

## Abbreviations

The following abbreviations are used in this manuscript:

IAV	influenza A virus
HPAI	highly pathogenic avian influenza
BSL-3	Biosafety Level 3
MDCK	Madin-Darby canine kidney
TCID_50_	50% tissue culture infectious dose
CA7	A/California/07/2009
VN1203	A/Vietnam/1203/2004
GY5096	A/Duck/Guiyang/5096/2013
SZ2400	A/Chicken/Shenzhen/2400/2013
SP378	A/Shenzhen/SP378/2015
HZ4258	A/Duck/Huzhou/4258/2013
AH1	A/Anhui/1/2013
SH2	A/Shanghai/2/2013
SP440	A/Guangdong/SP440/2017
WZ598	A/Chicken/Wenzhou/598/2013
JX346	A/Jiangxi-Donghu/346/2013
LPAI	low pathogenic avian influenza
SPF	specific-pathogen-free
SD	Sprague-Dawley
PBS	phosphate-buffered saline
dpi	day(s) post-inoculation
RDE	receptor-destroying enzyme
TRBCs	turkey red blood cells
HAI	hemagglutination inhibition
WHO	World Health Organization
IHC	immunohistochemical
NP	nucleoprotein
H&E	hematoxylin and eosin
SA	sialic acid
MAA	*Maackia amurensis* agglutinin
SNA	*Sambucus nigra* agglutinin
ANOVA	one-way analysis of variance
AUC	area under the curve
pdmH1N1	pandemic 2009 H1N1
mTEDCs	mouse tracheal epithelial cells
