# Supplementary material for: Experimental Infection of Rats with Influenza A Viruses: Implications for Murine Rodents in Influenza A Virus Ecology
Source: Viruses. 2025 Mar 29;17(4):495. doi: 10.3390/v17040495 (PMC12030792; doi:10.3390/v17040495)

Figure S1. Distribution of α2,3 and α2,6-linked sialosides in the respiratory tracts of mice. The α2,3- and α2,6-linked sialosides in the nasal turbinate (A, B, C), trachea (D, E, F) and lung (G, H, I) of naïve female C57BL/6 mice were detected with biotinylated *Maackia amurensis* agglutinin I or II (MAA I, MAA II) for α2,3-linked sialosides or *Sambucus nigra* agglutinin (SNA) for α2,6-linked sialosides. Both α2,3- and α2,6-linked sialosides were visible in brown. Scale bars represent 200 μm at 20× magnification in images A-F and 40× magnification in G-I.

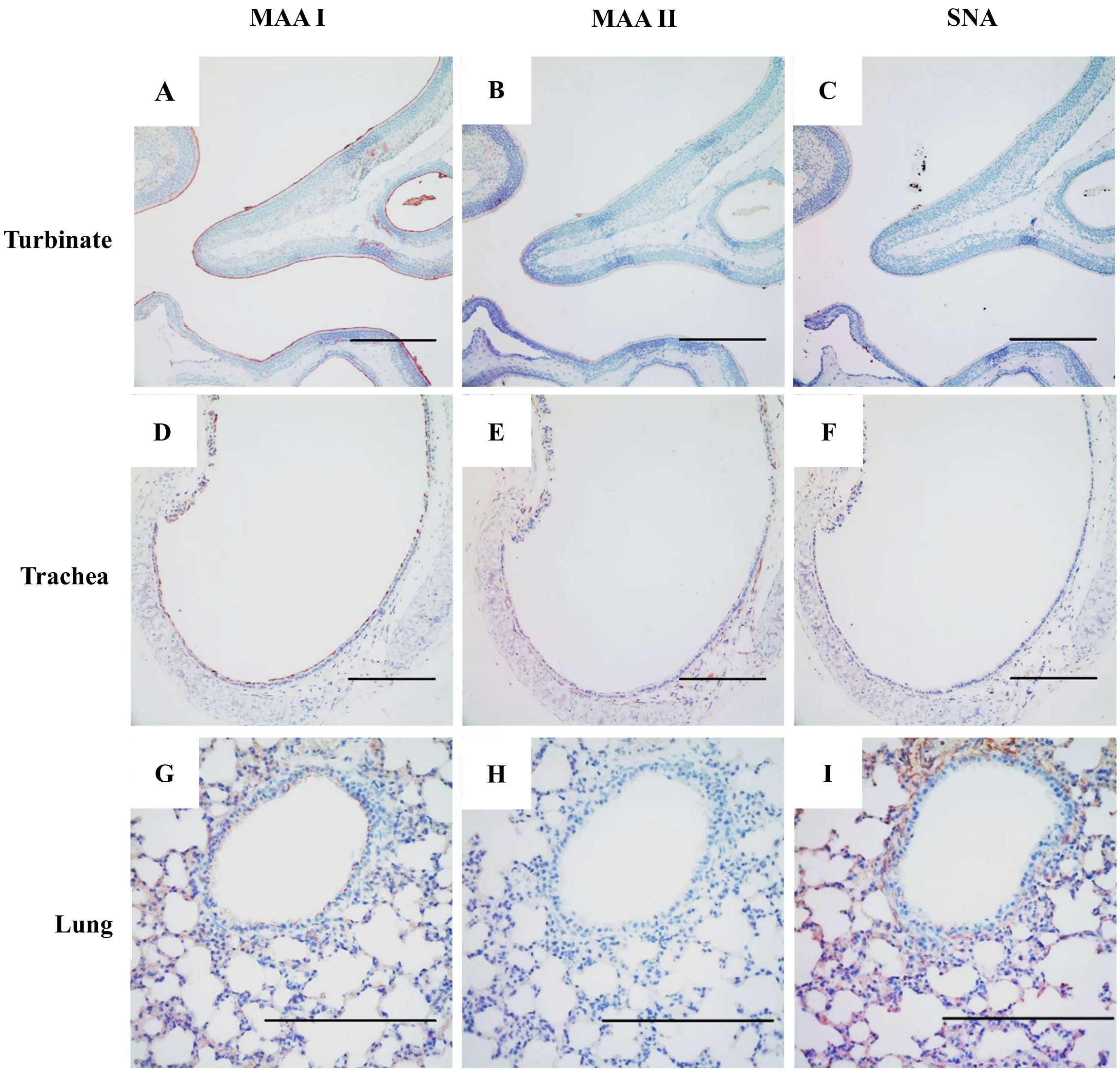

Supplement: Supplementary file 1 [file viruses-17-00495-s001.zip › Figure S1.pdf]
