# Supplementary material for: Experimental Infection of Rats with Influenza A Viruses: Implications for Murine Rodents in Influenza A Virus Ecology
Source: Viruses. 2025 Mar 29;17(4):495. doi: 10.3390/v17040495 (PMC12030792; doi:10.3390/v17040495)
Supplement: Supplementary file 1 [file viruses-17-00495-s001.zip › Table S1.pdf]

Table S1. Seroconversion in Sprague-Dawley (SD) rats inoculated with the tested influenza A viruses[a]

Virus inoculated	Subtype	Virus used for the Hemagglutination Inhibition (HAI) testing										
		CA7	VN1203	GY5096	SZ2400	SP378	HZ4258	AH1	SH2	SP440	WZ598	JX346
**PBS**	(Mock)	-[b]	-	-	-	-	-	-	-	-	-	-
**CA7**	H1N1	5/5 (1280)[c]	-	-	-	-	-	-	-	-	-	-
**VN1203**	H5N1	-	5/5 (80-320)	1/5 (10)	-	-	-	-	-	-	-	-
**GY5096**	H5N1	-	2/5 (20-40)	2/5 (10-20)	1/5 (20)	1/5 (20)	-	-	-	-	-	-
**SZ2400**	H5N6	-	-	-	3/5 (10-40)	-	2/5 (10)	-	-	-	-	-
**SP378**	H5N6	-	1/5 (<10)	-	-	3/5 (10)	-	-	-	-	-	-
**HZ4258**	H5N8	-	-	-	-	-	1/5 (10)	-	-	-	-	-
**AH1**	H7N9	-	-	-	-	-	-	5/5 (320-1280)	5/5 (640-1280)	5/5 (40-80)	-	-
**SH2**	H7N9	-	-	-	-	-	-	5/5 (80-320)	5/5 (320-640)	5/5 (40-80)	-	-
**SP440**	H7N9	-	-	-	-	-	-	5/5 (20-40)	5/5 (40-80)	5/5 (40-80)	-	-
**WZ598**	H9N2	-	-	-	-	-	-	-	-	-	5/5 (640-1280)	-
**JX346**	H10N8	-	-	-	-	-	-	-	-	-	-	5/5 (1280)

a, Groups of five SD rats were inoculated intranasally with PBS (mock) or 10^6 $TCID_{50}$ of each virus (see Table 1) and blood was collected at the end of the experiment (14 dpi).

b, -, Antibodies not detected.

c, Number of seropositive rats / Number of total rats (range of the HAI titers).
